# Strepchazolins A and B: Two New Alkaloids from a Marine *Streptomyces chartreusis* NA02069

**DOI:** 10.3390/md15080244

**Published:** 2017-08-02

**Authors:** Cheng-Long Yang, Yi-Shuang Wang, Cheng-Li Liu, Ying-Jie Zeng, Ping Cheng, Rui-Hua Jiao, Shi-Xiang Bao, Hui-Qin Huang, Ren-Xiang Tan, Hui-Ming Ge

**Affiliations:** 1 State Key Laboratory of Pharmaceutical Biotechnology, Institute of Functional Biomolecules, School of Life Sciences, Nanjing University, Nanjing 210023, China; yangchenglong1022@163.com (C.-L.Y.); 13529110064@163.com (Y.-S.W.); wendyliucl@163.com (C.-L.L.); 18013966315@163.com (Y.-J.Z.); chengp1209@126.com (P.C.); rhjiao@nju.edu.cn (R.-H.J.); 2 State Key Laboratory Cultivation Base for TCM Quality and Efficacy, Nanjing University of Chinese Medicine, Nanjing 210023, China; 3 Key Laboratory of Biology and Genetic Resources of Tropical Crops of Ministry of Agriculture, Institute of Tropical Biosciences and Biotechnology, Chinese Academy of Tropical Agricultural Sciences, Haikou 571101, China; baoshixiang@itbb.org.cn

**Keywords:** alkaloid, strepchazolin, streptazolin, *Streptomyces charteus*, marine natural product, antimicrobial activity, acetylcholinesterase inhibitory activity

## Abstract

Two new alkaloids, strepchazolins A (**1**) and B (**2**), together with a previously reported compound, streptazolin (**3**), were isolated from a marine actinomycete, *Streptomyces chartreusis* NA02069, collected in the Coast of Hainan Island, China. The structures of new compounds were determined by extensive NMR, mass spectroscopic and X-ray crystallographic analysis, as well as modified Mosher’s method. Compound **1** showed weak anti-*Bacillus subtilis* activity with the MIC value of 64.0 μM, and weak inhibitory activity against acetylcholinesterase (AChE) in vitro with IC_50_ value of 50.6 μM, while its diastereoisomer, Compound **2**, is almost inactive.

## 1. Introduction

**Figure 1:**
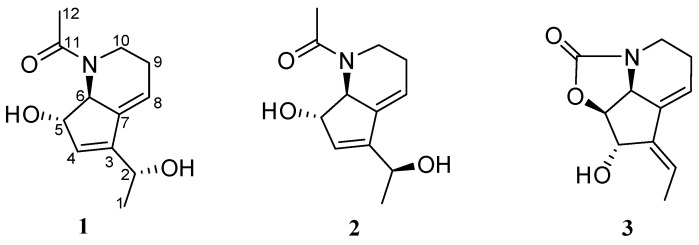
Structure of compounds (**1**–**3**) isolated from *S. chartreusis* NA02069.

Research conducted over the past several decades in marine microorganisms has clearly indicated that the ocean is a great resource of microorganisms for producing structurally diverse and biologically active secondary metabolites due to their unique environment [1]. Among all known microbes, the filamentous bacteria in the order Actinomycetales are considered as the most prolific source for producing secondary metabolites [2,3,4], consisting of unusual polyketides, nonribosomal peptides, terpenoids, alkaloids, lipids and others. For instance, marinomycins A–D were isolated from a marine sediment culture of *Marinispora* sp. CNQ-140 showing significant antibacterial activities against drug-resistant pathogens, and selective cancer cell cytotoxicities against six melanoma cell lines [5]. Lajollamycins, a family of nitro group-bearing spiro-β-lactone-γ-lactams were produced by a marine-derived *Streptomyces* sp. [6]. Salinosporamide, a potent proteasome inhibitor with an unusal β-lactone moiety isolated from a marine *Salinospora tropica* has been entered human clinical trial for the treatment of multiple myeloma [7]. In our search for new bioactive compounds from marine microorganisms, we isolated actinomycete strains from a marine sediment sample in the Coast of Hainan Island of China. During the screening, we found one of the isolated strains, NA02069, was designated as *Streptomyces chartreusis* based on its 16s rDNA sequence. *S. chartreusis* has been previously reported to produce chartreusin, chrymutasins, and calcimycin [8,9], all of which belong to the family of polyketides. In order to enrich the diversity of the compounds from this strain, we became interested in *S. chartreusis* NA02069 to find whether different compounds can be produced. In this study, we report the isolation and structure elucidation of two new (**1**) and (**2**) and a known (**3**) alkaloid compounds (Figure 1). Compounds **1** and **2** were evaluated for their antimicrobial and acetylcholinesterase inhibitory activities.

## 2. Results

### 2.1. Taxonomy and Phylogenetic Analysis of the Strain NA02069

**Figure 2:**
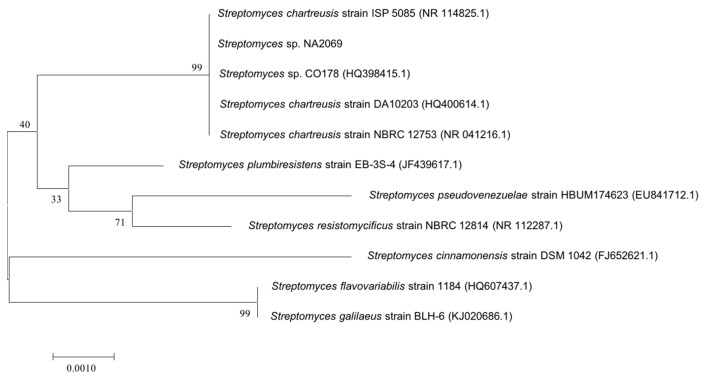
Neighbor-joining tree based on 16s rDNA sequence of strain NA02069.

The 16s rDNA sequence of strain NA02069 was amplified by polymerase chain reaction (PCR) and sequenced. After alignment analysis using Basic Logic Alignment Search Tool (BLAST), strain NA02069 showed 100% identities (1352 bp) to a subclade, which also included the type strains of *Streptomyces chartreusis* ISP 5085, *S. chartreusis* DA10203, and *S. chartreusis* NBRC 12753 (Figure 2). Thus, the strain was assgined as *Streptomyces chartreusis* NA02069 [10].

### 2.2. Isolation and Structure Elucidation

The actinomycete strain was cultured at 28 °C with 130 rpm agitation in YEME medium (20 L) medium. After 10 days, the filtrate of the fermented culture broth was extracted repeatedly with ethyl acetate three times to afford a brown crude extract, which was subjected to repeated column chromatography (CC) over silica-gel, Sephadex LH-20, octadecylsilane (ODS) and high performance liquid chromatography (HPLC) to yield two new alkaloids, strepchazolins A (**1**), B (**2**), and a known Compound (**3**). The known compound was identified as streptazolin by comparison of their spectroscopic data with those in the literature [11].

**Figure 3:**
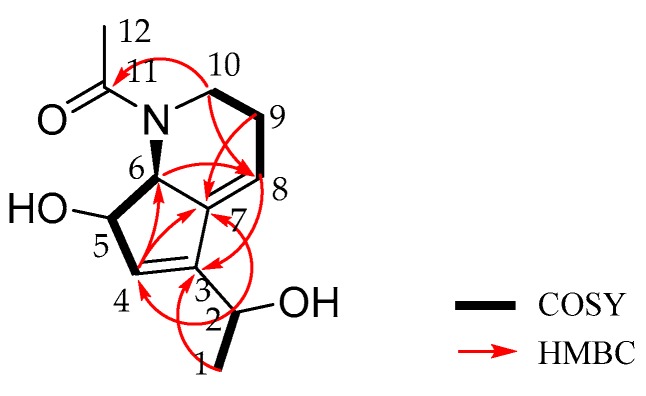
The Key 2D NMR correlations of **1**.

Strepchazolin A (**1**) was obtained as a pale yellow crystal. Its molecular formula was determined as C_12_H_17_NO_3_ on the basis of the sodium-adduct high-resolution ESIMS ion at *m/z* 246.1116 [M + Na]^+^, (calcd for C_12_H_17_NO_3_Na, 246.1101) along with the ^13^C NMR data (Table 1). The ^1^H NMR spectrum of **1** in methanol-*d*_4_ displayed two double-bond protons (*δ*_H_ 5.85 and 6.05), three oxygen/nitrogen substituted methine protons (*δ*_H_ 4.51, 4.56, and 4.31), four methylene protons (*δ*_H_ 3.16, 3.83, 2.30 and 2.30), and six methyl protons in two methyl groups (*δ*_H_ 1.38 and 2.19). The ^13^C NMR spectrum showed one carbonyl carbon (*δ*_C_ 175.4), four resonances in the double bond region (*δ*_C_ 148.5, 140.7, 130.3, and 117.8), three oxygen/nitrogen substituted carbons (*δ*_C_ 81.0, 68.4, and 64.7), and four sp^3^-hybrized quaternary carbons (*δ*_C_ 46.2, 25.8, 22.8, and 22.6). Further analysis of its HSQC spectrum assigned all signals for ^1^H-^13^C one bond correlations. The ^1^H-^1^H COSY spectrum of H-1 (*δ*_H_ 1.38)/H-2 (*δ*_H_ 4.51); H-4 (*δ*_H_ 5.85)/H-5 (*δ*_H_ 4.56)/H-6 (*δ*_H_ 4.31); and H-8 (*δ*_H_ 6.05)/H-9 (*δ*_H_ 2.30)/H-10 (*δ*_H_ 3.16 and 3.83) revealed three spin systems are present in **1** (Figure 3). The HMBC correlations of H-1/C-3, H-2/C-4, H-6/C-8, H-9/C-7 suggested the above three spin systems were connected through C-2-C-3-C-4, and C-6-C-7-C-8, respectively. Moreover, the HMBC correlations of H-2/C-7, H-4/C-7, as well as H-8/C-3 revealed the connectivity between C-3-C-7, revealing the presence of a cyclopentene moiety. The HMBC correlations of H-6/C-8 and H-10/C-8 together with their typical nitrogen substituted chemical shifts for *δ*_C-6_ 68.4/*δ*_H-6_ 4.31 and *δ*_C-10_ 46.2/*δ*_H-10a_ 3.83 and *δ*_H-10b_ 3.16 indicated the presence of a tetrahydropyridine moiety. Finally, the HMBC correlation of H-10 and H-12 with carbonyl carbon C-11 revealing that an acetyl group was anchored at the N-atom. The remaining two hydroxyl groups were connected at C-2 (*δ*_C_ 64.7) and C-5 (*δ*_C_ 81.0) on the basis of their chemical shifts. Thus, the planar structure of **1** was established.

**Figure 4:**
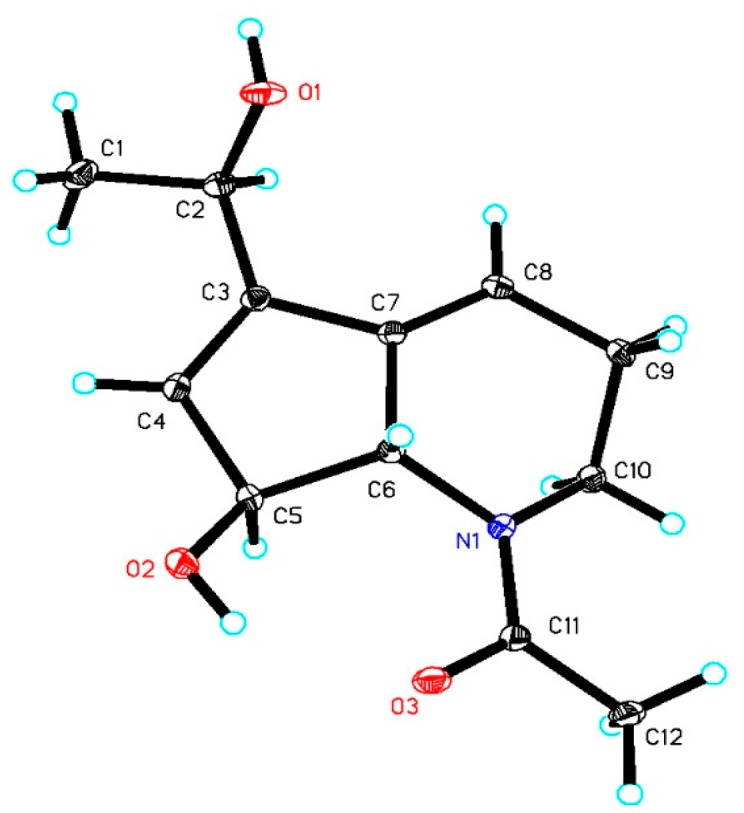
X-ray crystal structure of compound **1**.

The full structure and absolute configurations at all chiral centers of **1** were assigned based on the X-ray crystallographic analysis. A high-quality single crystal was obtained from slow evaporation of compound **1** in acetone/MeOH solutions at low temperature. The X-ray crystallography data measured in a Cu Kα radiation in low temperature fully confirmed the proposed structure and determined the absolute configuration of **1** as 2*R*, 5*S*, and 6*S* (Figure 4).

**Figure 5:**
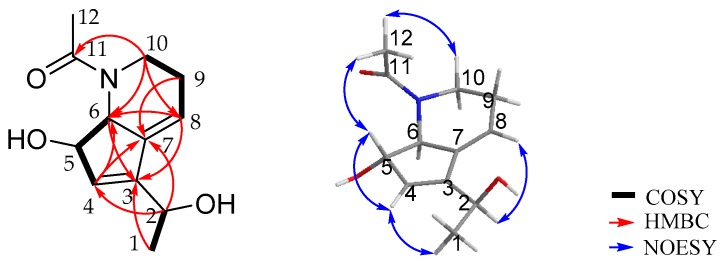
The Key 2D NMR correlaitons of **2**.

Strepchazolin B (**2**) was obtained as a pale yellow oil, which analyzed for the same molecular formula, C_12_H_17_NO_3_, as **1** by HRESIMS (obsd [M + Na]^+^ at *m/z* 246.1103, calcd for C_12_H_17_NO_3_Na 246.1101) in combination with interpretation of ^1^H and ^13^C NMR data (Table 1). The ^13^C NMR data of **2** were almost identical to those of **1**, however the ^1^H NMR spectra between **1** and **2** were slightly different (Figure S17). Further interpretation of HSQC, HMBC and ^1^H-^1^H COSY spectral data (Figure 5) of **2** established that the planar structure of compound **2** is identical to that of **1**.

**Figure 6:**
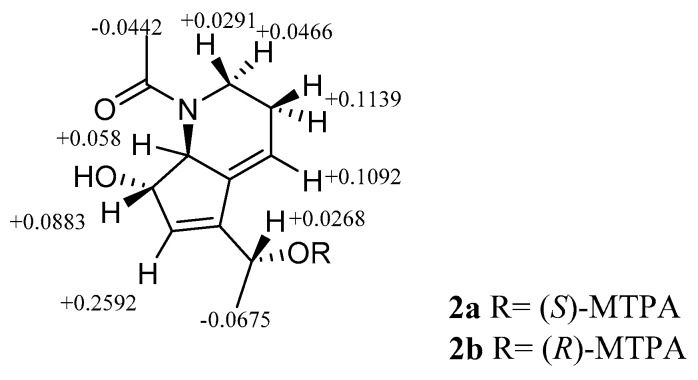
^1^H NMR Δ*δ_S-R_* values in ppm between the (*S*)- and (*R*)-MTPA esters **2a** and **2b** in methanol*-d*_4_.

In the NOESY spectrum of **2** (Figure 5), the NOE correlation between H-5 and H-12 demonstrated that H-5 and H-6 possessed β and α-orientations, respectively, consistent with those observed in **1**. The chemical shifts for H-5/C-5 and H-6/C-6 in **2** are the same with those in **1**, which suggest configurations of C-5 and C-6 in **2** are the same to **1**. The absolute configuration of C-2 in compound **2** was determined by a modified Mosher’s method [12,13]. Using low temperature control of the esterify reaction (MTPA-Cl/pyridine), we were able to regioselectively esterified the C-2 hydroxyl group in **2**. The ^1^H chemical shifts of **2a** and **2b** were assigned by analyzing their HSQC and ^1^H-^1^H COSY NMR spectra. The Δ*δ* values between (*S*) and (*R*)-MTPA esters demonstrated that C-2 possessed an *S*-configuration (Figure 6), which is opposite to that in compound **1** as expected.

### 2.3. Bioactivities of Strepchazolins 

As a primary screen for antibacterial and acetylcholinesterase inhibitory activities, the results were shown in Table 2 and Table 3. Compound **1** exhibited weak antibacterial activity against *Bacillus subtilis* with its MIC value of 64.0 μM, and weak acetylcholinesterase inhibitory activity with IC_50_ value of 50.6 μM. However, compound **2**, the diastereoisomer of **1**, is almost inactive (Table 2 and Table 3).

## 3. Experimental Section

### 3.1. General Methods

Optical rotation was measured in MeOH on a Rudolph Research Analytical Autopol IV automatic polarimeter. Mass spectra were acquired on an Agilent 6250 TOF LC-MS instrument equipped with an electrospray ionization (ESI) probe operating in positive-ion mode with direct infusion. NMR experiments were accomplished in Methanol-*d*_4_ on a Bruker DRX-600 spectrometer with ^1^H and ^13^C nuclei observed at 600 and 150 MHz, respectively, with TMS or solvent signal adopted as internal standards. Silica gel (200–300 mesh) for CC (column chromatography) and GF254 (10–20 µm) for TLC (thin layer chromatography) were purchased from Qingdao Marine Chemical Company, China. Sephadex LH-20 were purchased from GE Biotech, USA. Semi-preparative reverse phase-high performance liquid chromatography (RP-HPLCs) were accomplished on an Eclipse XDB-C18 column (5 μM, 250 × 9.4 mm) from Agilent Technologies Inc. USA (Santa Clara, CA, USA). All chemicals used in the study were of analytical grade and HPLC grade.

### 3.2. Strain Isolation and Cultivation

*S. chartreusis* NA02069 was isolated from a sediment from the coast of Hainan Island, China. The purified strain was cultured in modified 38^#^ medium plate (consisting 4 g yeast extract, 4 g glucose, 5 g malt extract, 1 mL multi-vitamins, 20 g agar, 27 g sea salt in 1 L water) at 28 °C for 4 days. After 2 days of incubation in 10 flasks each containing 100 mL Tryptone Soya Broth medium (Weigh 30 g to 1 L of water) at 28 °C with 130 rpm agitation, the seed cultures were used to inoculate 100 flasks each containing 200 mL YEME medium (consisting 4 g yeast extract, 4 g glucose, 10 g malt extract, 27 g sea salt in 1 L water).

### 3.3. Isolation of Compounds ***1***, ***2*** and ***3***

After 10 days cultivation, the filtrate (20 L) of the fermented culture broth was extracted three times using ethyl acetate. The organic extract was enriched in vacuo to yield 3.2 g of a brown thick oil material. The extract was subjected to column chromatography (CC) over silica gel fractionated with 500 mL mixtures of petroleum ether-ethyl acetate (*v/v*, 100:0, 100:2, 100:4, 100:10, 100:50, 0:100), CH_2_Cl_2_-MeOH (*v/v*, 100:0, 100:2, 100:4, 100:10, 100:50, 0:100) to afford 17 fractions (04-1→04-17 collected according to TLC monitoring). 04-5 (0.5 g) was subsequently separated by ODS chromatography with H_2_O-MeOH (*v/v*, 70:30, 60:40, 50:50, 40:60, 30:70, 0:100) to afford 8 fractions (04-5-1→04-5-8 collected according to TLC monitorings). Finally, compounds **1** (11.2 mg), **2** (9.4 mg), **3** (6.7 mg) were eluted as pure compounds using semi-preparative reverse-phase HPLC (2 mL/min, detector UV λ_max_ 254 nm, MeOH/H_2_O 38:62).

Compound **1**: Pale yellow crystal; [α]${}_{D}^{25}$ −68° (*c* 0.10, MeOH); m.p.: 125–126 °C; UV (MeOH) λ_max_ (log *ε*): 217 (0.791), 281 (0.391) nm. HR-ESI-MS (*m*/*z*): 246.1116 [M + Na]^+^ (calcd. for C_12_H_17_NO_3_Na, 246.1101); ^1^H and ^13^C NMR data are listed in Table 1.

Compound **2**: pale yellow oil; [α]${}_{D}^{25}$ −26° (*c* 0.1, MeOH); UV (MeOH) λ_max_ (log ε): 217 (0.714), 281 (0.224) nm. HR-ESI-MS (*m*/*z*): 246.1103 [M + Na]^+^ (calcd. for C_12_H_17_NO_3_Na, 246.1101); ^1^H and ^13^C NMR data are listed in Table 1.

### 3.4. Crystal Data of ***1***

Crystal data of compound **1** was collected on an Agilent Xcalibur Nova single-crystal diffractometer using Cu Kα radiation in low temperature. The structures were solved by direct methods using SHELXS-97 and refined by means of full-matrix least squares. Crystal data of compound **1** have been deposited with the Cambridge Crystallographic Data Centre as supplementary publication CCDC 1557150.

Crystal data for **1**: molecular formula C_12_H_17_NO_3_, *M*_r_ = 223.26, monoclinic crystals, *a* = 4.37020 (10) Å, *b* = 14.0896 (4) Å, *c* = 9.1364 (2) Å, *Z* = 2, *μ* = 0.779 mm^−1^ and *F* (000) = 240; Crystal dimensions: 0.180 × 0.150 × 0.100 mm^3^, Volume = 558.42 (2) Å^3^, *R* (reflections) = 0.0185 (1573), *R* indices [I > 2 sigma(I)] *R*_1_ = 0.0278, *wR*_2_ = 0.0739.

### 3.5. Formation of the (S)- and (R)-MTPA Esters of ***2***

To a solution of **2** (0.3 mg, 1.3 μmol) in anhydrous pyridine (0.15 mL), (*R*)-(−)-MTPA chloride (1.6 mg, 6.5 μmol) was added, and the reaction mixture was stirred at 0 °C for 1 h. Methanol (0.3 mL) was added to the reaction mixture to terminate the reaction. The solvent was evaporated under reduced pressure, and the residue was purified by HPLC using Acetonitrile-H_2_O (65:35→90:10) as the eluent to afford (*S*)-MTPA ester **2a** (0.1 mg) as a colorless oil. **2** (0.3 mg, 1.3 μmol) and (*S*)-(+)-MTPA chloride (1.6 mg, 6.5 μmol) were treated with the same procedure to afford (*R*)-MTPA ester **2b** (0.1 mg) as a colorless oil.

*S-*MTPA ester (**2a**): colorless oil; HR-ESI-MS (*m*/*z*): 462.1486 [M + Na]^+^ (calcd. for C_22_H_24_F_3_NO_5_Na, 462.1499); ^1^H NMR data are listed in Table S1 of Supplementary Materials.

*R-*MTPA ester (**2b**): colorless oil; HR-ESI-MS (*m*/*z*): 462.1500 [M + Na]^+^ (calcd. for C_22_H_24_F_3_NO_5_Na, 462.1499); ^1^H NMR data are listed in Table S1 of Supplementary Materials.

### 3.6. Antibacterial Assay

Following the described procedure [14], the antibacterial activities of compounds **1** and **2** were tested in 96-well microplates. The tested bacterial strain, Bacillus subtilis, was preserved in State Key Laboratory of Pharmaceutical Biotechnology, Nanjing University, China. The strain was grow on a MHB solid medium and then cultured 24 h in liquid medium. The testing system consists of 50 μL diluted bacterial solution (~105 CFU/mL), 49 μL fresh medium and 1 μL tested compound. The microplate was vibrated for homogenizing the mixture and then incubated at 37 °C for 24 h. MIC (Minimal Inhibitory Concentration) accounts for the magnitude of the antibacterial activity.

### 3.7. Bioactivity Assay

The acetylcholinesterase inhibitory activities were assayed according to a method reported in the literature [15]. Briefly, compounds **1** and **2** were dissolved in DMSO and diluted to different concentrations using 0.1 M phosphate buffer (pH 8.0). The enzymatic reaction was triggered by adding 20 μL of 3.33 mM 5,5′-dithio-bis (2-nitrobenzoic acid) (DTNB, Sigma-Aldrich, St. Louis, MO, USA) to the reaction system (consisting of 2 μL of the tested sample solution, 20 μL of 0.35 U/mL AChE solution and 20 μL of 3.33 mM 5,5′-dithio-bis (2-nitrobenzoic acid) (DTNB) and 138 μL of phosphate buffer). Reaction substances were detected through Microplate Reader by measuring the absorbance at 410 nm. Meanwhile, Huperzine A and DMSO were used as positive and negative controls, respectively. Each test was performed in triplicate. Then IC_50_ of each compound was calculated by SPSS 16.0 [16].

## 4. Conclusions

In summary, two new alkaloids, strepchazolins A (**1**) and B (**2**), as well as a known compound streptazolin (**3**) were isolated from a marine actinomycete *S. chartreusis* NA02069. Their structures were determined using a combination of HRESIMS and NMR spectroscopic and X-ray crystallographic data together with modified Mosher’s method. Compounds **1** showed weak anti-*Bacillus subtilis* activity and weak inhibitory activity against acetylcholinesterase, while its diastereoisomer, compound **2**, is almost inactive. The research described here further indicates that marine actinomycetes are a rich source for new bioactive natural products that could lead to the discovery of new drugs or drug leads.

## Figures and Tables

**Table 1:** ^1^H and ^13^C NMR (600 and 150 MHz in methanol-*d*_4_) data for compounds **1** and **2**.

No.	1		2	
	*δ*c, Type	*δ*_H_ (*J* in Hz)	*δ*_C_, Type	*δ*_H_ (*J* in Hz)
1	22.6, CH_3_	1.38, d (6.5)	22.7, CH_3_	1.36, d (6.5)
2	64.7, CH	4.51, q (6.5)	64.8, CH	4.55, q (6.5)
3	148.5, C		149.2, C	
4	130.3, CH	5.85, brs	129.0, CH	5.86, brs
5	81.0, CH	4.56, m	81.0, CH	4.56, m
6	68.4, CH	4.31, brs	68.4, CH	4.30, brs
7	140.7, C		140.9, C	
8	117.8, CH	6.05, m	117.2, CH	5.96, m
9	25.8, CH_2_	2.30, m	25.8, CH_2_	2.30, m
10	46.2, CH_2_	3.16, td (12.2,2.9)	46.2, CH_2_	3.16, td (12.2,2.9)
		3.83, dt (12.2,2.9)		3.83, dt (12.2,2.9)
11	175.4, C		175.4, C	
12	22.8, CH_3_	2.19, s	22.8, CH_3_	2.20, s

**Table 2:** The antimicrobial MIC (μM) of compounds **1** and **2**.

	1	2	Rifampicin
*Bacillus subtilis*	64.0	>128.0	1.0

**Table 3:** In vitro acetylcholinesterase (AChE) inhibitory activity of compounds **1** and **2**.

Compound	1	2	Huperzine A
IC_50_ (μM)	50.6	>100.0	4.3

## Supplementary Materials

The following are available online at www.mdpi.com/1660-3397/15/8/244/s1, Supplementary Materials including HRESIMS spectra, 1D and 2D NMR spectra, tabulated NMR data for **2a** and **2b**.
