# Supplementary material for: Strepchazolins A and B: Two New Alkaloids from a Marine Streptomyces chartreusis NA02069
Source: Mar Drugs. 2017 Aug 2;15(8):244. doi: 10.3390/md15080244 (PMC5577599; doi:10.3390/md15080244)
Supplement: Supplementary file 1 [file marinedrugs-15-00244-s001.pdf]

SUPPORTING INFORMATION

Strepchazolins A and B: Two New Alkaloids from a Marine *Streptomyces chartreusis* NA02069

Cheng Long Yang[1], Yi Shuang Wang[1], Cheng Li Liu[1], Ying Jie Zeng[1], Ping Cheng[1], Rui Hua Jiao[1], Shixiang Bao[3], Huiqin Huang[3,*], Ren Xiang Tan[1,2,*], and Hui Ming Ge[1,*]

[1] State Key Laboratory of Pharmaceutical Biotechnology, Institute of Functional Biomolecules, School of Life Sciences, Nanjing University, Nanjing 210023, China;

[2] State Key Laboratory Cultivation Base for TCM Quality and Efficacy, Nanjing University of Chinese Medicine, Nanjing 210023, China;

[3] Institute of Tropical Biosciences and Biotechnology, Key Laboratory of Biology and Genetic Resources of Tropical Crops of Ministry of Agriculture, Chinese Academy of Tropical Agricultural Sciences, Haikou 571101, China;

Supplementary Materials

Table S1. NMR data (methanol-*d*4, ^{1}H 600 MHz, ^{13}C 150 MHz) for **2a** and **2b** 2
Figure S1. HRESIMS of compound **1** 2
Figure S2. ^{1}H NMR spectrum for **1** in methanol-*d*4 (600 MHz) 3
Figure S3. ^{13}C NMR spectrum for **1** in methanol-*d*4 (150 MHz) 3
Figure S4. DEPT spectrum for **1** in methanol-*d*4 (150 MHz) 4
Figure S5. COSY spectrum for **1** in methanol-*d*4 (600 MHz) 4
Figure S6. HSQC spectrum for **1** in methanol-*d*4 (600 MHz) 5
Figure S7. HMBC spectrum for **1** in methanol-*d*4 (600 MHz) 5
Figure S8. NOESY spectrum for **1** in methanol-*d*4 (600 MHz) 6
Figure S9. HRESIMS of compound **2** 6
Figure S10. ^{1}H NMR spectrum for **2** in methanol-*d*4 (600 MHz) 7
Figure S11. ^{13}C NMR spectrum for **2** in methanol-*d*4 (150 MHz) 7
Figure S12. DEPT spectrum for **2** in methanol-*d*4 (150 MHz) 8
Figure S13. COSY spectrum for **2** in methanol-*d*4 (600 MHz) 9
Figure S14. HSQC spectrum for **2** in methanol-*d*4 (600 MHz) 9
Figure S15. HMBC spectrum for **2** in methanol-*d*4 (600 MHz) 10
Figure S16. NOESY spectrum for **2** in methanol-*d*4 (600 MHz) 10
Figure S17. Comparison of the ^{1}H NMR spectra of compounds **1** and **2** 11
Figure S18. Comparison of the ^{13}C NMR spectra of compounds **1** and **2** 11

Table S1. NMR data (methanol-d_4, ^{1}H 600 MHz, ^{13}C 150 MHz) for **2a** and **2b**

**Position**	$\delta_{H\text{-}2a}$	$\delta_{H\text{-}2b}$	$\Delta\delta_H=\delta_{H\text{-}2a}-\delta_{H\text{-}2b}$
1	1.4690	1.5365	-0.0675
2	5.8839	5.8571	+0.0268
3	-	-	-
4	5.8746	5.6154	+0.2592
5	4.5786	4.4903	+0.0883
6	4.3181	4.2601	+0.058
7	-	-	-
8	6.0121	5.9029	+0.1092
9	2.2954	2.1815	+0.1139
10	3.8312,3.1716	3.8021,3.1250	+0.0291, +0.0466
11	-	-	-
12	1.9911	2.0353	-0.0442

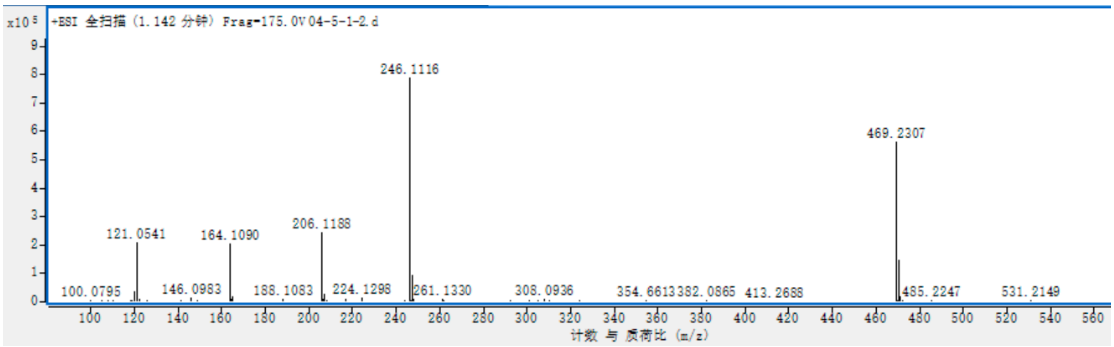

Figure S1. HRESIMS of compound **1**

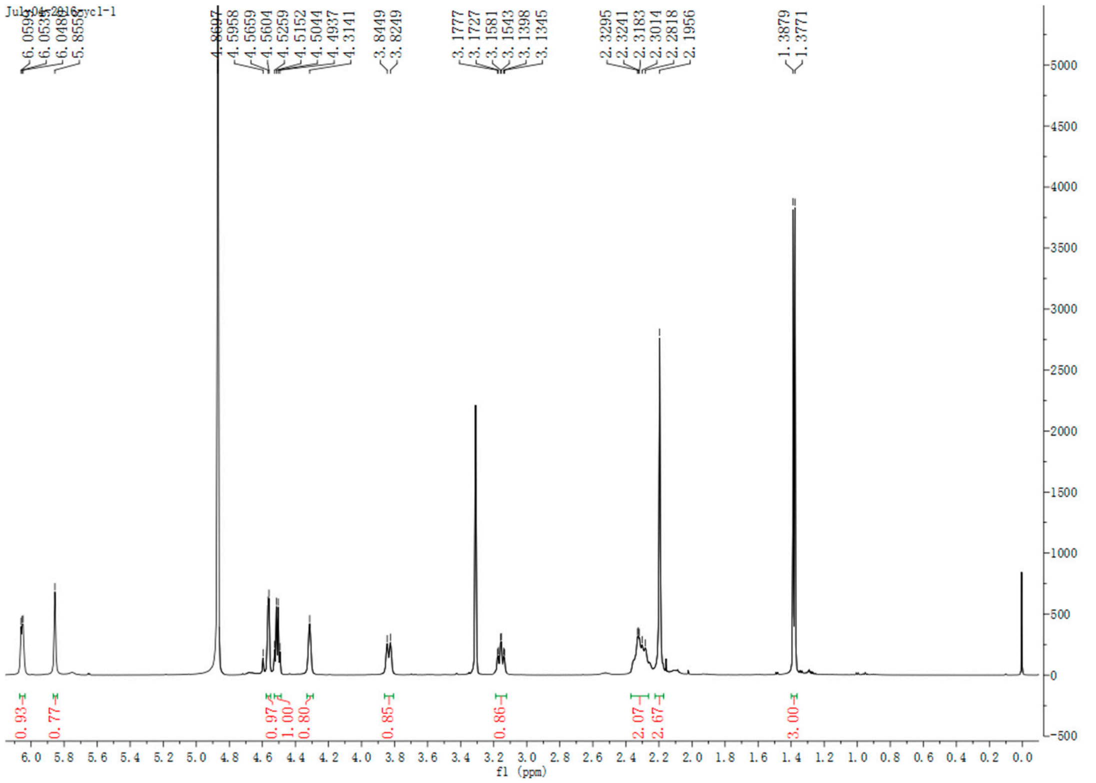

Figure S2. ^{1}H NMR spectrum for **1** in methanol-d_4 (600 MHz)

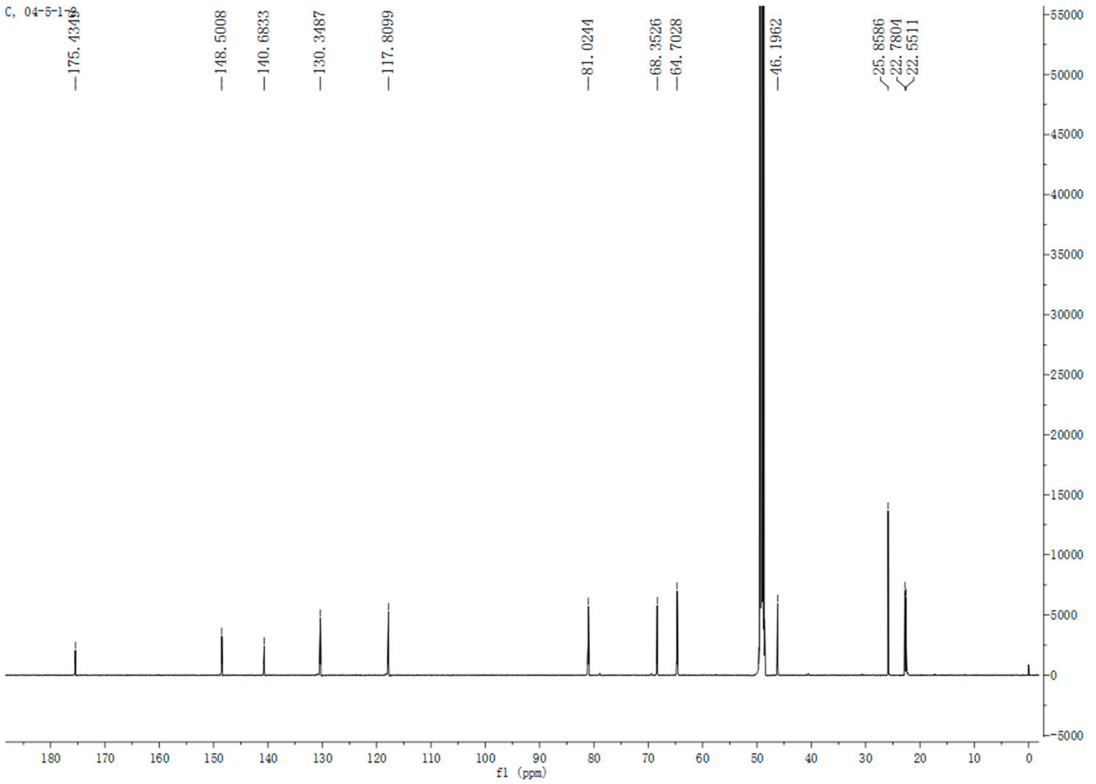

Figure S3. ^{13}C NMR spectrum for **1** in methanol-d_4 (150 MHz)

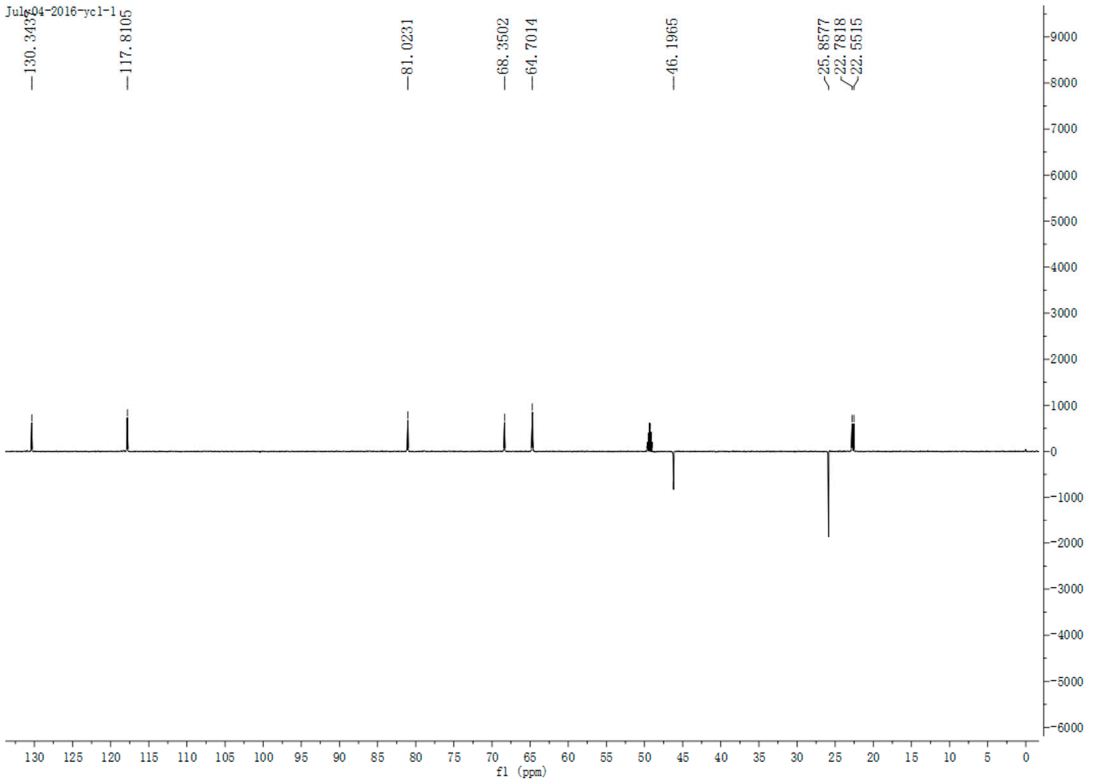

Figure S4. DEPT spectrum for **1** in methanol-d_4 (150 MHz)

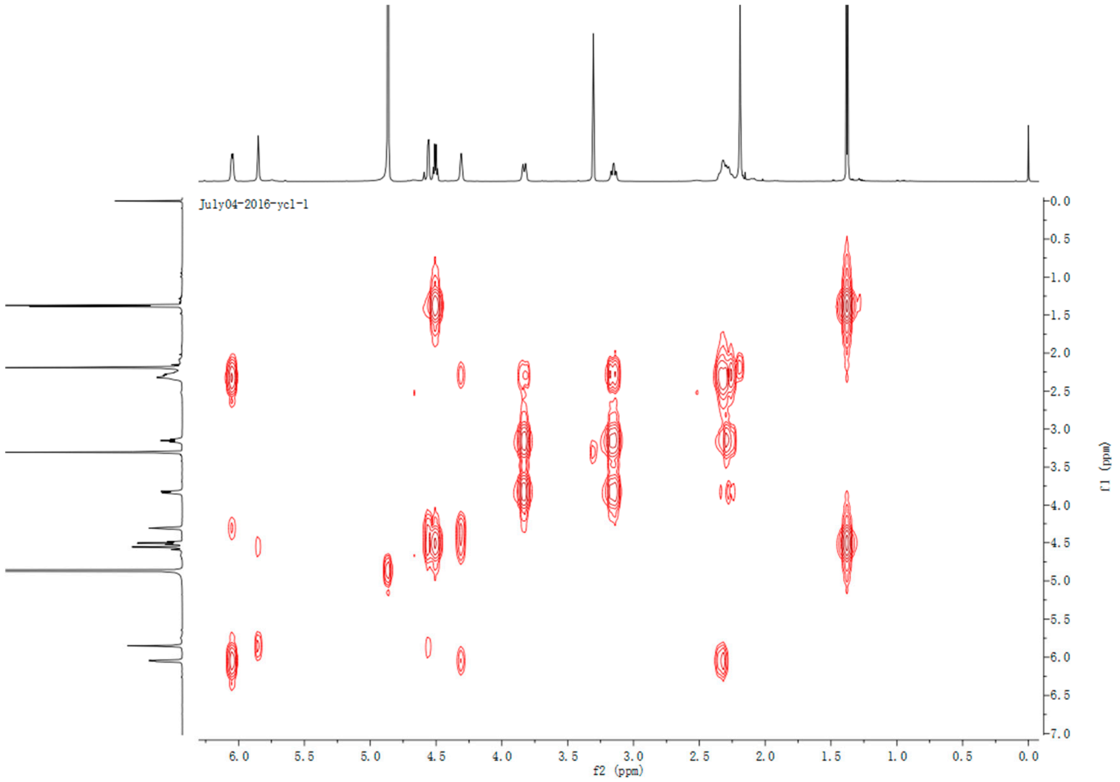

Figure S5. COSY spectrum for **1** in methanol-d_4 (600 MHz)

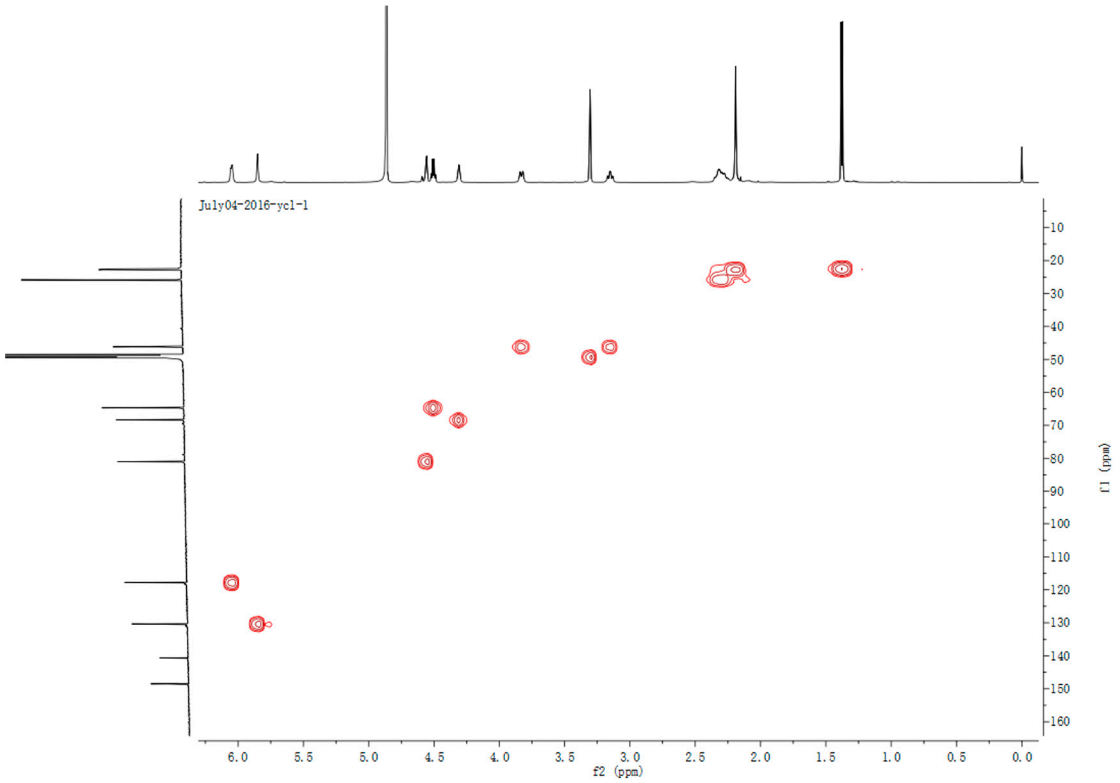

Figure S6. HSQC spectrum for **1** in methanol-d_4 (600 MHz)

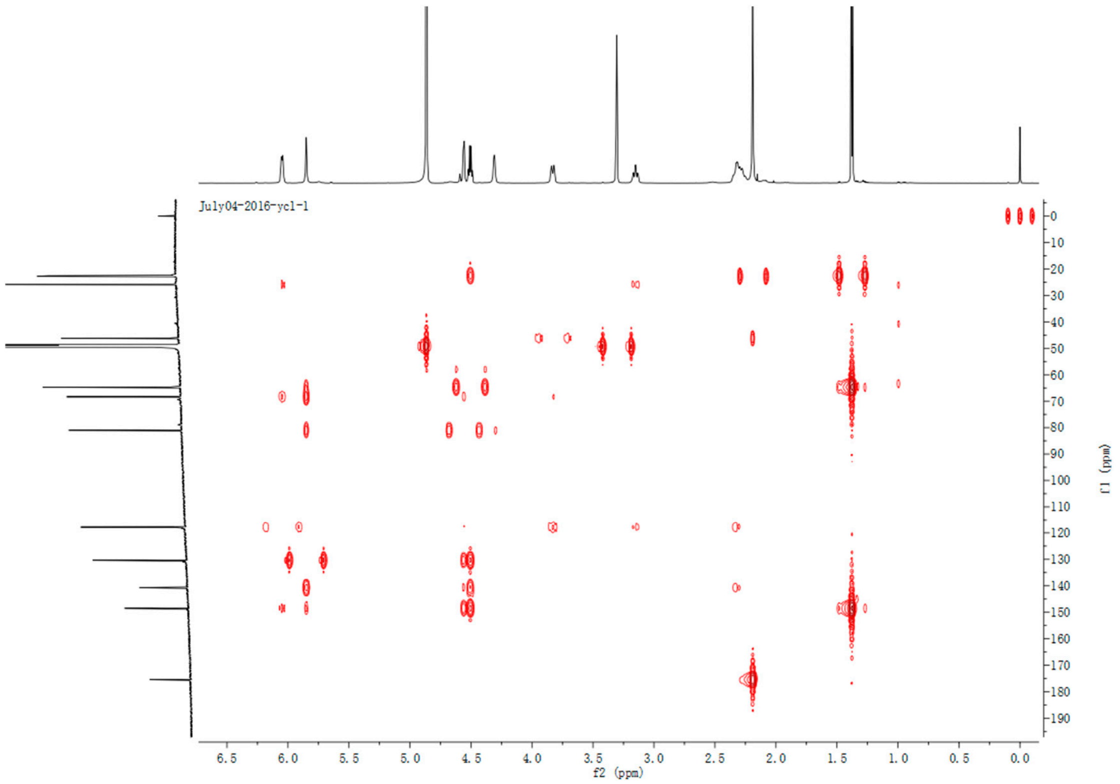

Figure S7. HMBC spectrum for **1** in methanol-d_4 (600 MHz)

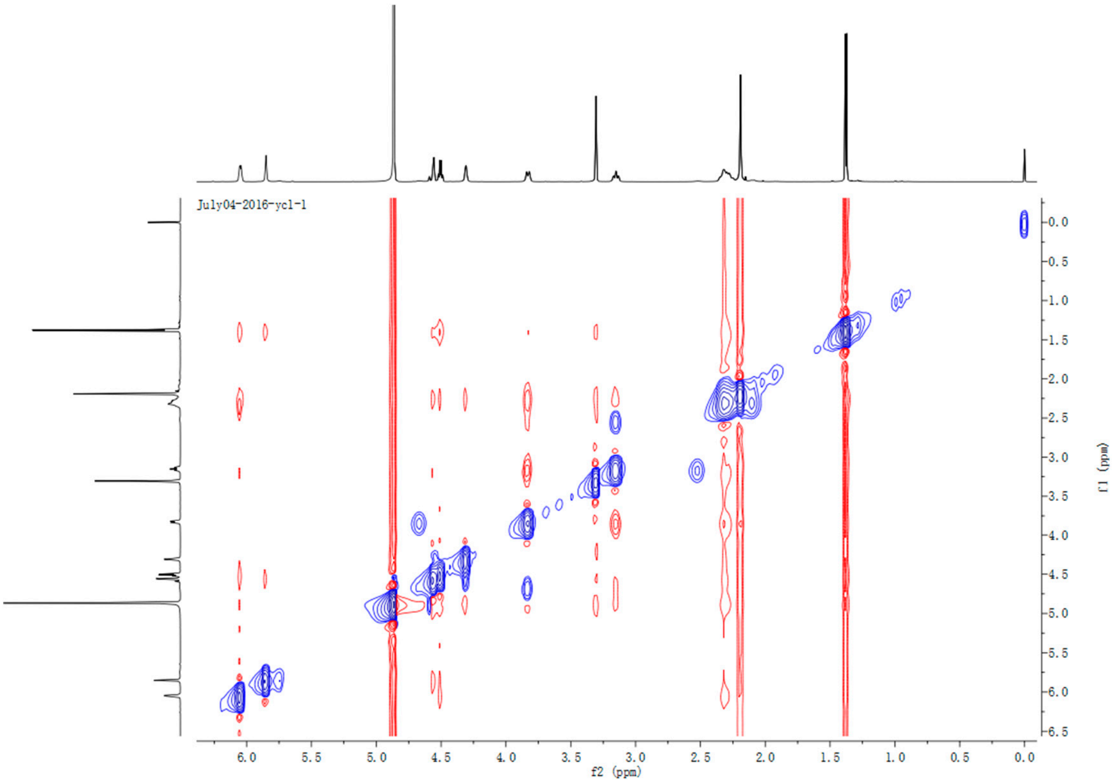

Figure S8. NOESY spectrum for **1** in methanol-d_4 (600 MHz)

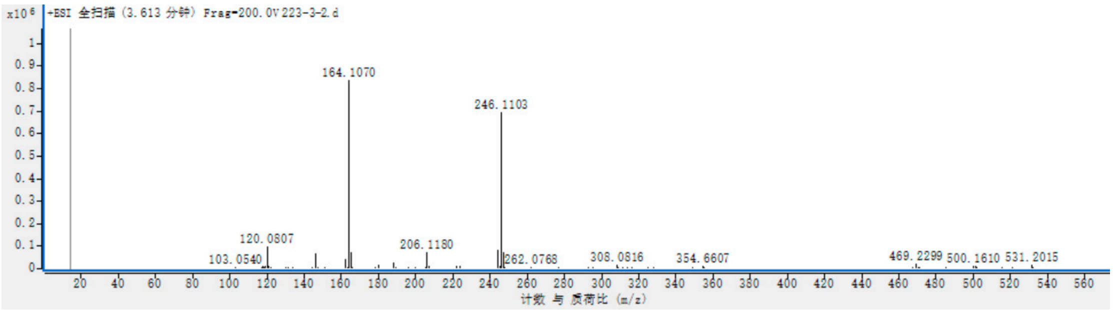

Figure S9. HRESIMS of compound **2**

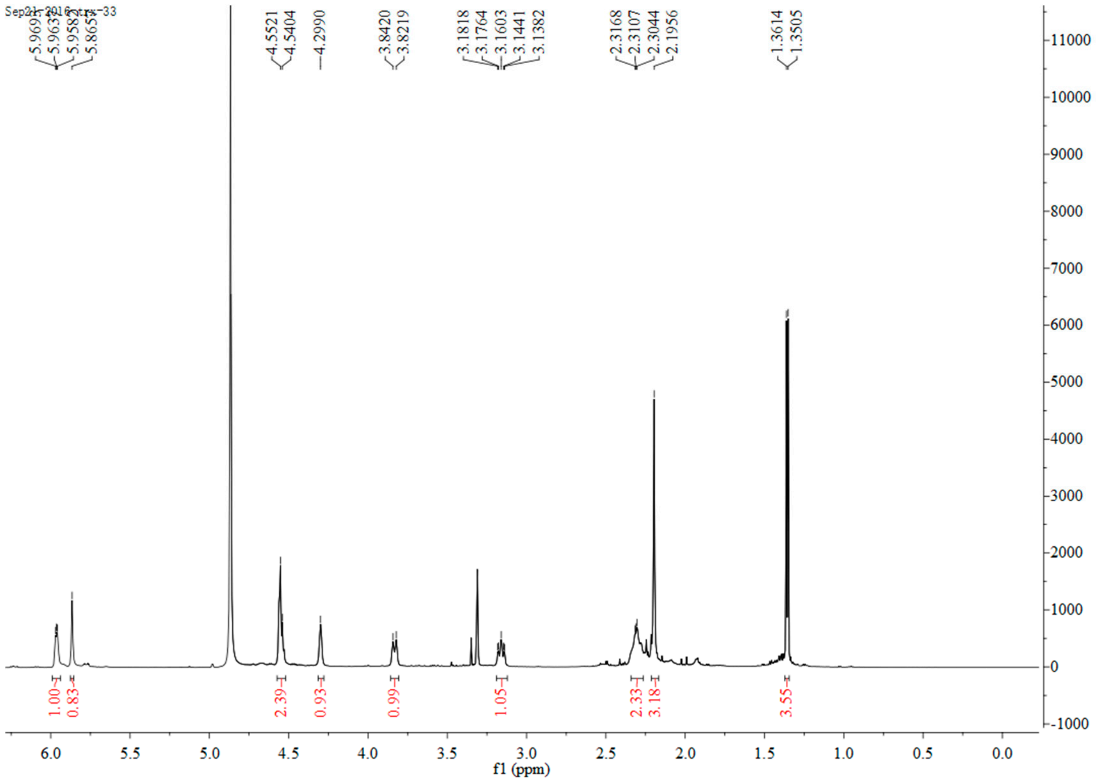

Figure S10. ^{1}H NMR spectrum for **2** in methanol-d_4 (600 MHz)

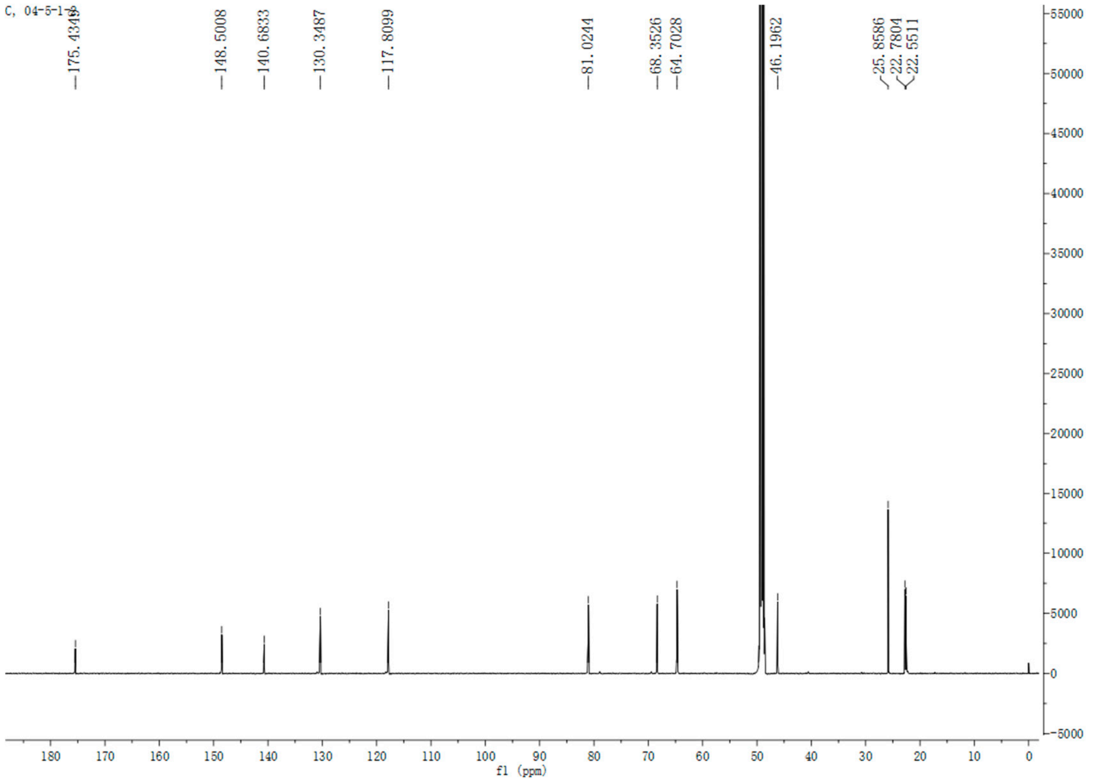

Figure S11. ^{13}C NMR spectrum for **2** in methanol-d_4 (150 MHz)

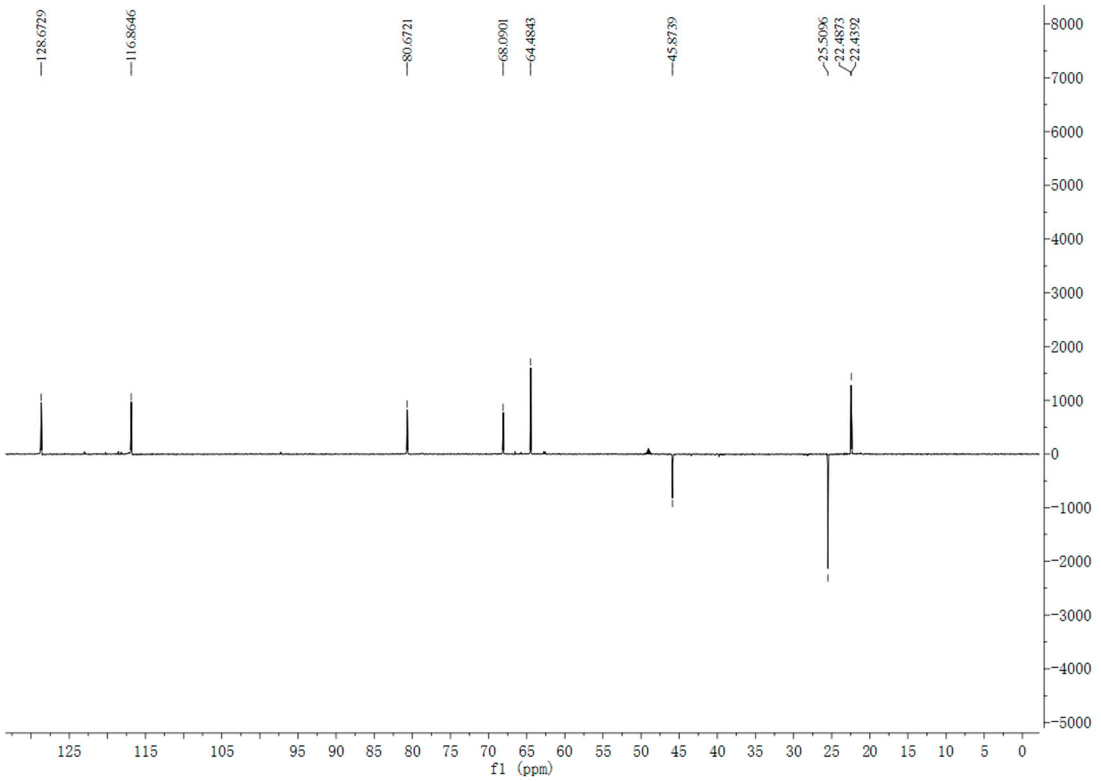

Figure S12. DEPT spectrum for **2** in methanol-d_4 (150 MHz)

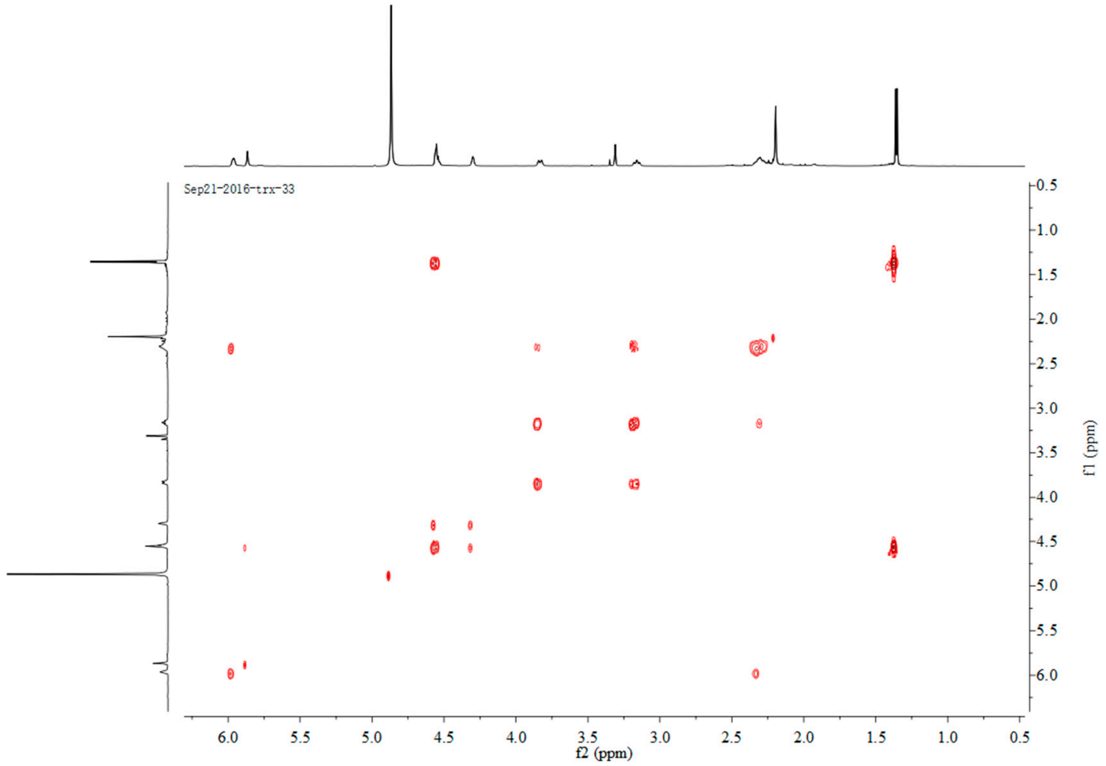

Figure S13. COSY spectrum for **2** in methanol-d_4 (600 MHz)

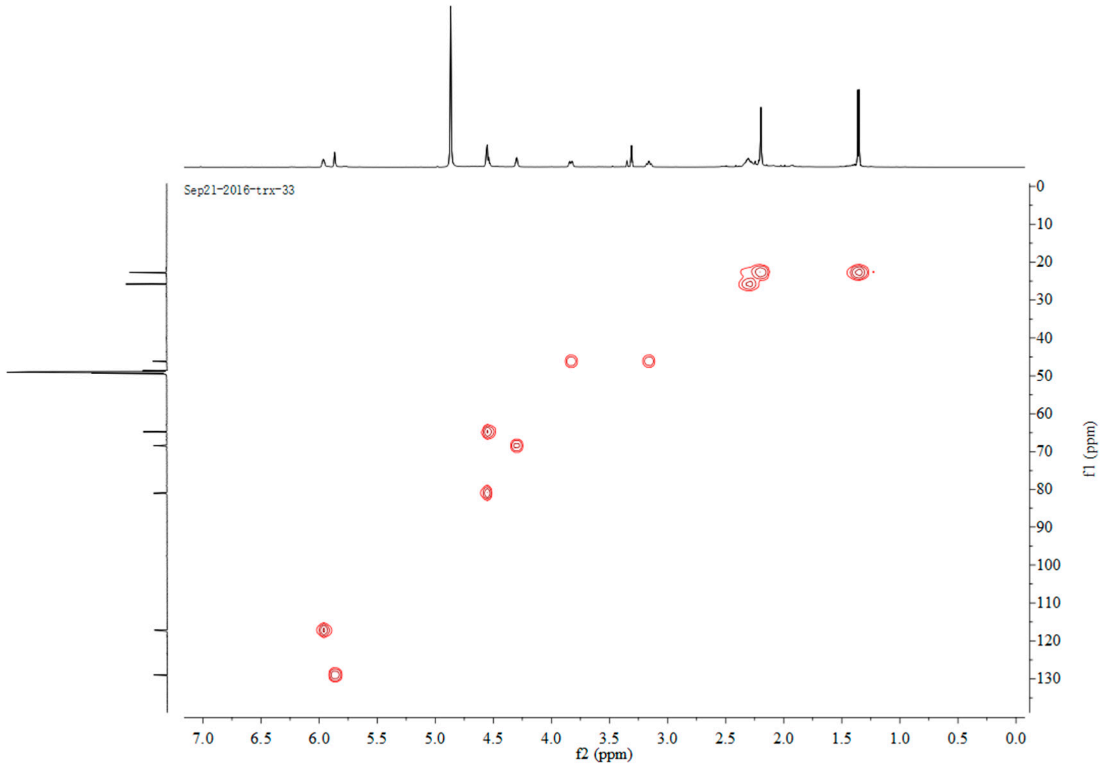

Figure S14. HSQC spectrum for **2** in methanol-d_4 (600 MHz)

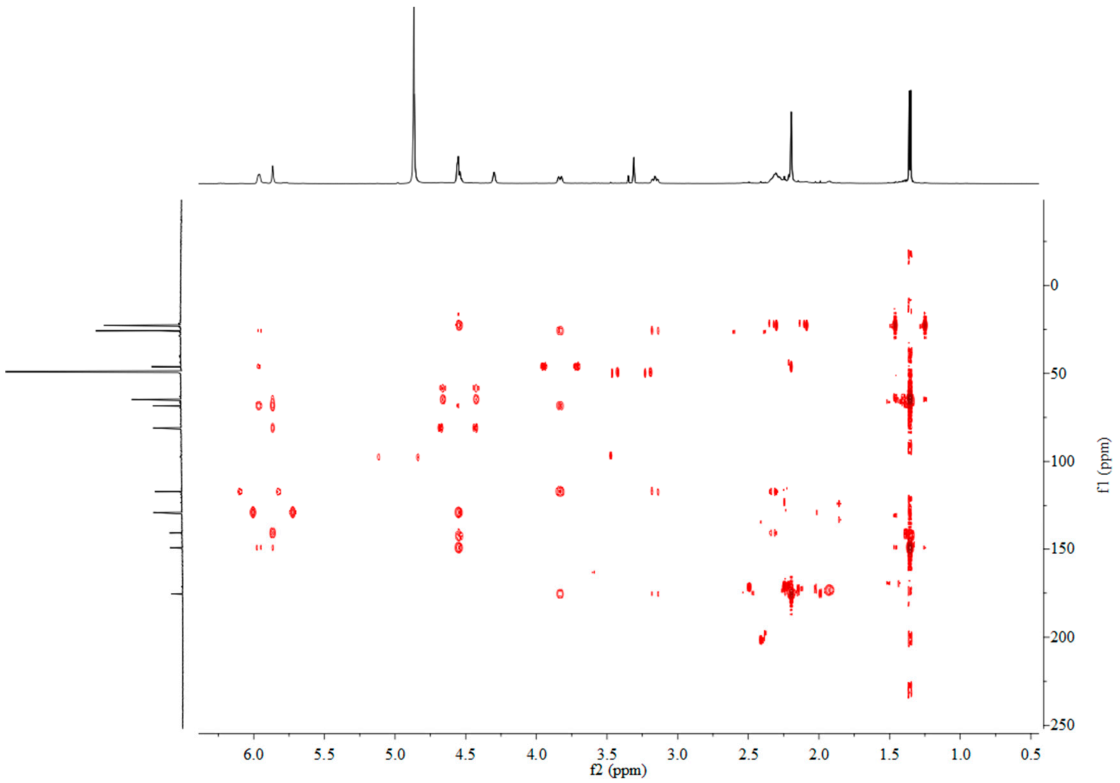

Figure S15. HMBC spectrum for **2** in methanol-d_4 (600 MHz)

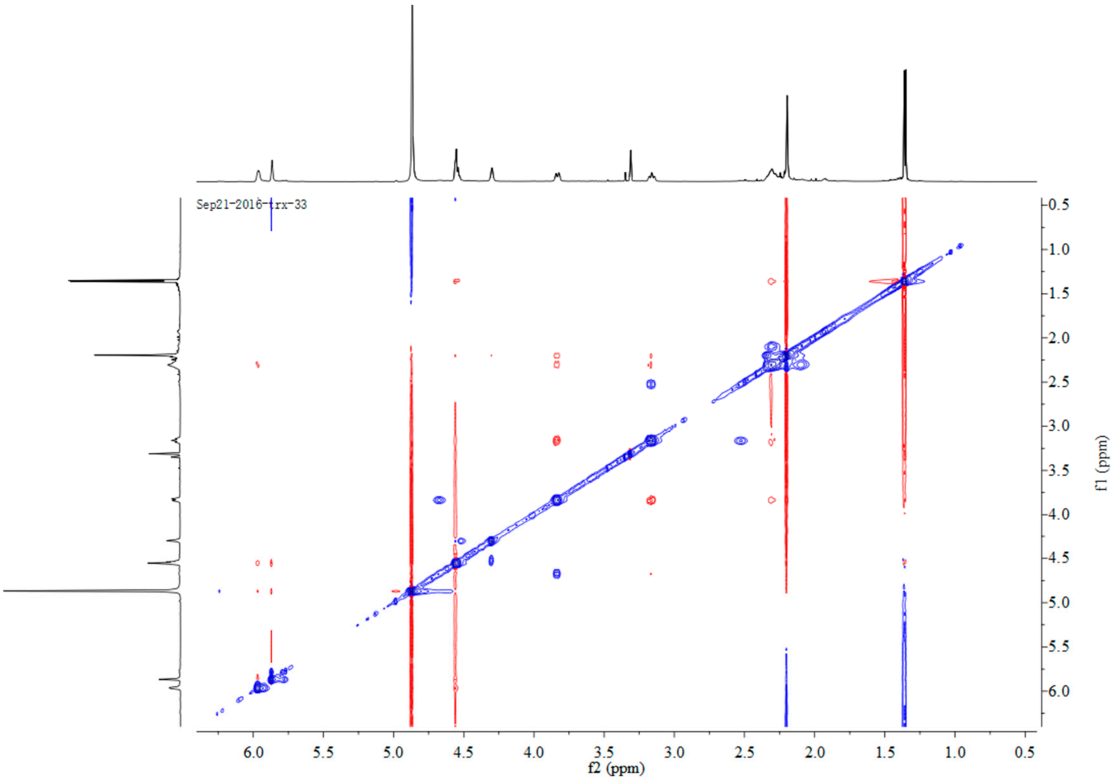

Figure S16. NOESY spectrum for **2** in methanol-d_4 (600 MHz)

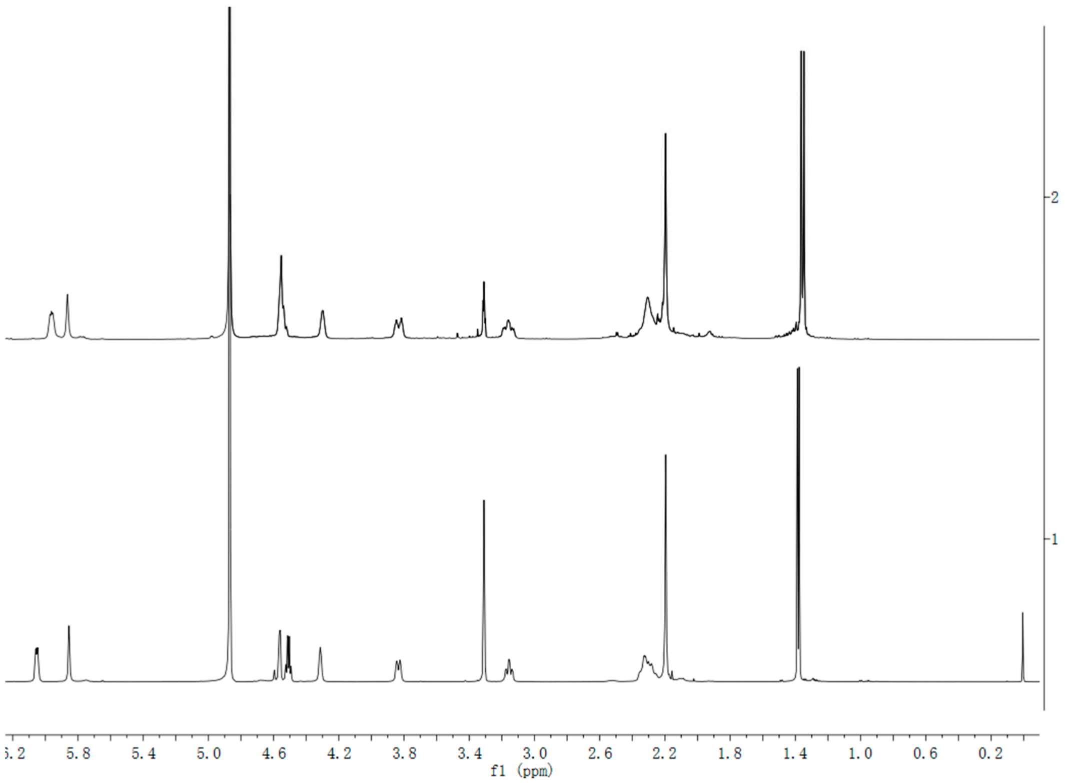

Figure S17. Comparison of the ^{1}H NMR spectra of compounds **1** and **2**

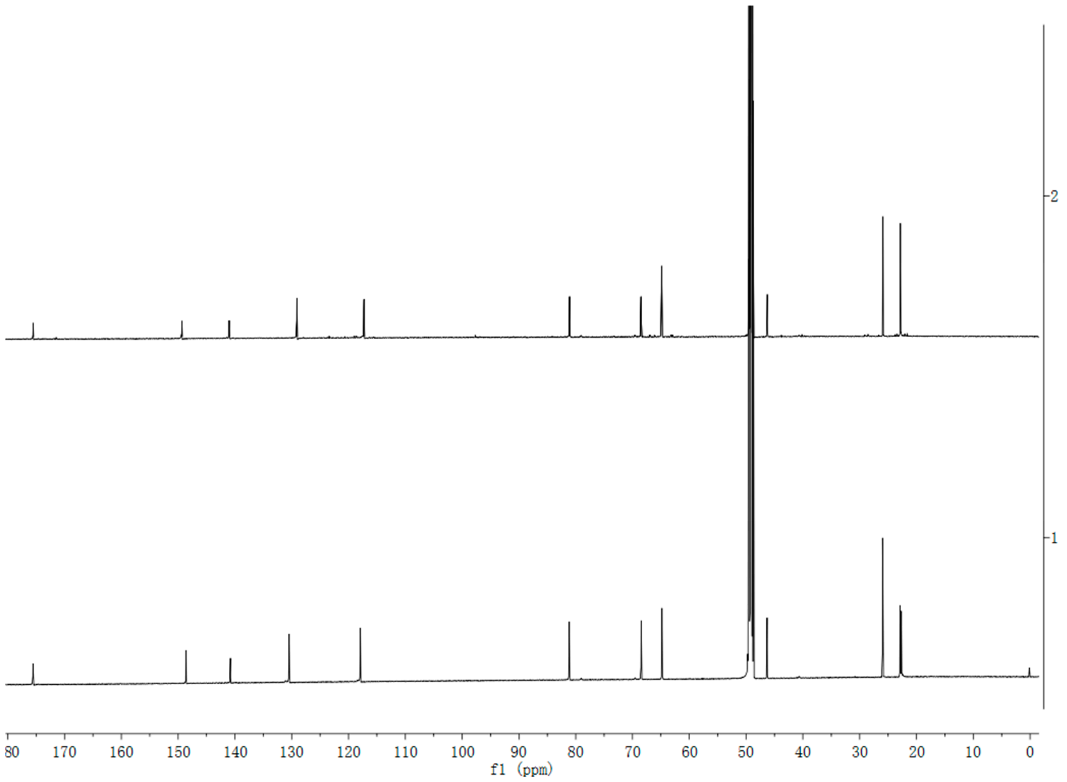

Figure S18. Comparison of the ^{13}C NMR spectra of compounds **1** and **2**
